# Seeing Double: Exploring the Phenomenology of Self-Reported Absence of Rivalry in Bistable Pictures

**DOI:** 10.3389/fnhum.2017.00301

**Published:** 2017-06-09

**Authors:** Elisa Filevich, Maxi Becker, Yuan-hao Wu, Simone Kühn

**Affiliations:** ^1^Max Planck Institute for Human Development, Lifespan PsychologyBerlin, Germany; ^2^Department of Psychology, Humboldt Universität zu BerlinBerlin, Germany; ^3^Bernstein Center for Computational Neuroscience BerlinBerlin, Germany; ^4^Berlin School of Mind and Brain, Humboldt-Universität zu BerlinBerlin, Germany; ^5^Clinic and Policlinic for Psychiatry and Psychotherapy, University Clinic Hamburg-EppendorfHamburg, Germany; ^6^Neurocomputation and Neuroimaging Unit, Department of Education and Psychology, Freie Universität BerlinBerlin, Germany

**Keywords:** bistable perception, rivalry, fMRI, perceptual switches, FFA, PPA

## Abstract

Ambiguous images such as Rubin’s vase-face can be interpreted in at least two different ways. These interpretations are typically taken to be mutually exclusive, and ambiguous images have thus served as models of perceptual competition. Here, we present data that challenges this view. In an online survey, we found that a large proportion of people within the general population reported that the two percepts were not competing but could be perceived simultaneously. Of those who reported that they could see both percepts simultaneously, we invited 17 participants to take part in a functional magnetic resonance imaging (fMRI) experiment. In the scanner, participants saw images that could be interpreted as either a landscape or a face and reported at every point in time whether they perceived predominantly the face, the landscape, or both simultaneously. We explored behavioral and neurophysiological (with fMRI) correlates of the reported subjective experience of entertaining two percepts simultaneously by comparing them to those of the simple percepts (i.e., face or landscape). First, by comparing percept durations, we found that the simultaneous state was as stable as the two other percepts. Second, by measuring blood-oxygen-level-dependent (BOLD) signal levels within the fusiform face area (FFA), occipital face area (OFA) and parahippocampal place area (PPA), we found evidence from objective data that confirmed the subjective reports. While the results in FFA and OFA were not conclusive, in PPA, BOLD signal levels during subjective reports of perceiving both a landscape and a face were lower than the BOLD signal levels associated with reports of perceiving a landscape (and, in turn, reports of seeing a landscape were associated with greater BOLD signal levels than reports of seeing a face, thus suggesting that BOLD signal levels in PPA are a valid correlate of subjective experience in this task). In sum, the objective measures suggest that entertaining two percepts simultaneously in mind can be regarded as a distinct (mixed) perceptual state. We argue with these results that a more central role of subjective report in cognitive neuroscience is sometimes warranted.

## Introduction

Ambiguous images, such as the famous Rubin vase (Rubin, 1915), can be interpreted in two different ways and are often used as examples of perceptual competition. These images are powerful tools to probe the neural bases of awareness because they lead to a situation where the content of awareness varies over time, in spite of no changes in visual stimulation (Crick and Koch, 1998; Blake et al., 2014; Giles et al., 2016). It is generally assumed that only one of the two possible interpretations is a conscious percept at any given point in time (Rubin, 2003). In the particular case of Rubin’s vase, the two possible percepts (a vase or two faces) are defined by a common edge. According to the principles of Gestalt psychology, this common edge necessarily demarcates the beginning or end of the figure or ground, which is why each part of the figure can only be interpreted as *either* figure or ground.

One interesting case challenges this view. In his book “Sampling Inner Experience in Disturbed Affect” Hurlburt (1993) reported a series of direct experience samples (Hurlburt and Heavey, 2006), where a patient described her pristine inner experience as often consisting in multiple simultaneous percepts, represented in the same imagined location. Hurlburt then examined more in detail the phenomenology of this perceptual superposition by using the Rubin’s vase figure (p. 208):

“… I then sketched Rubin’s well-known ambiguous figure of the two faces/vase. What do you see? I see two faces in profile, and a candlestick or vase or something in the middle. Do you see them both at the same time, or alternating, the faces and then the vase and then the faces, etc.? I see them both at the same time. Try to see the faces. Can you do that? Yes, but the vase is there also. […] I then turned my notepad, on which I had sketched the ambiguous figure for Fran, back towards me so that I could record my observations. The unintended result of this was that the faces/vase drawing was now turned obliquely on its side when Fran viewed it. “Oh”, she said, “now I see what you meant! It’s alternating now”, which meant that she experienced herself as focusing first on the faces, then on the vase, etc. After that, she could easily comprehend my questions about change of focus. …”

This intriguing report contrasts with the widespread notion that the two percepts in a bistable image are strictly alternating and instead suggests that they can in principle be superimposed in mental space and time.

Some previous work has considered situations in which two percepts coexist. A prime example of this situation is the phenomenon of traveling waves in binocular rivalry (Wilson et al., 2001; Arnold et al., 2009). During these transitional states, observers typically report that they see a “traveling wave”, in which one of the two competing percepts dominates only locally at first, as in a patch, and then this dominance starts to spread slowly over the whole percept. Traveling waves have been a topic of interest in their own right (for a review and discussion, see Brascamp et al., 2015) and studying their dependency on stimuli parameters has been a useful approach to understand the organization of the visual cortex (e.g., Bressloff and Webber, 2012) and its role in perceptual alternations (e.g., Lee et al., 2007). Besides traveling waves in binocular rivalry, mixed states in monocular stimuli have also been described. For example, Knapen et al. (2011) described their participants’ reported subjective experience of mixed, transitional states in apparent motion produced by a dot-quartet and a rotating spiral paradigm. Importantly, however, traveling waves and other mixed percepts are often considered transitional, and not stable, states. While perceptual dominance spreads in a finite (non-zero) period of time, one stimulus is always considered dominant. In contrast, the psychiatric patient with a diagnosis of Borderline Personality disorder described by Hurlburt (1993) reported that perceiving the two alternative interpretations of the image simultaneously was the most stable state. Interestingly, we carried out informal interviews and found that many people amongst the general population reported to have the ability (and in fact, the preference) to simultaneously perceive the two possible interpretations of a bistable image. Because these reports go counter to the widespread notion of perceptual competition, we sought to formally characterize this phenomenon.

**Figure 1:**
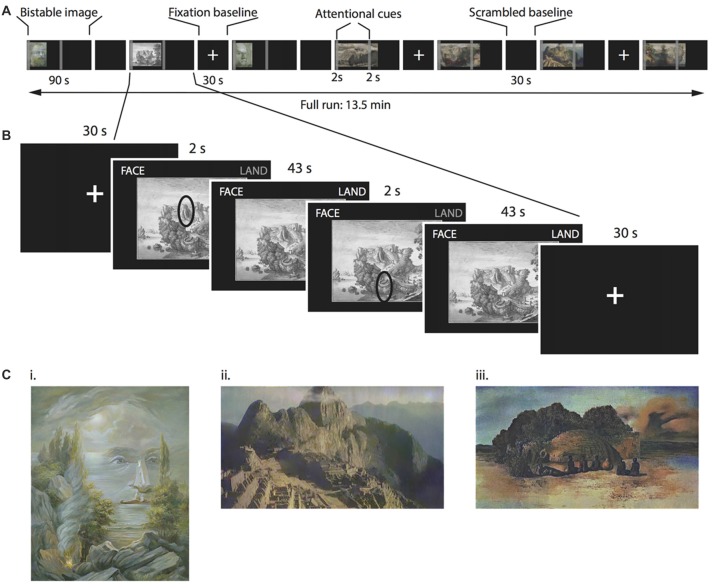
Multi-stable perception task. **(A)** Timeline for a single functional magnetic resonance imaging (fMRI) run. The order of figures was randomized between participants. **(B)** Detail of one multi-stable image. Each multi-stable image was displayed for 90 s, split into two 45-s periods. During each of these 45-s periods, we asked participants to attend to different parts of the image, indicated by a cue that appeared on the screen for 2 s. Participants continuously indicated with a button press whether the dominant percept was the face, the landscape or both percepts simultaneously. **(C)** Examples of the bistable images used as stimuli. **(i)** “Aivazovsky”, © Oleg Shupliak (http://www.art.ber.te.ua/), **(ii)** Anonymous modified picture from Machu Picchu and **(iii)** “Paranoiac Visage” by Salvador Dali, © VG Bild-Kunst, Bonn 2017.

The Rubin vase image used by Hurlburt and reported in the passage above is just one example of the many kinds of visual stimuli that can induce bistability—the alternation between two plausible perceptual interpretations of a stimulus which are regarded to be mutually exclusive (Long and Toppino, 2004; Schwartz et al., 2012). Bistability can arise at early stages of visual processing. For instance, binocular rivalry arises when conflicting information is delivered to each eye. This conflict is resolved before any of the two stimuli enter awareness (Frässle et al., 2014; Zou et al., 2016) allowing the information presented to only one of the two eyes to enter consciousness at any point in time. Apart from the exceptions described above, the transitional states between two percepts where the two stimuli are briefly mixed are seen as unstable and are usually discarded from analyses (e.g., Haynes et al., 2005; Frässle et al., 2014; but see Genç et al., 2015). Other bistable images do not rely on interocular conflict. Out of the wider range of examples, we consider here three categories of bistable visual stimuli, based on the kind of perceptual alternation that they allow for. First, the Rubin vase depends on a figure-ground distinction. Here, perceptual switches occur when either one of two parts of a figure is interpreted as the figure, automatically assigning the role of background to the other part (Rubin, 2003). In a second kind of bistable images, the background and figure remain always the same but the figure itself is ambiguous and can be reinterpreted in one of at least two ways, like in the famous duck-rabbit image example (Brugger, 1999). A third and final kind of bistable stimuli requires a switch between attending to the global and local aspects of an image. In these images, the details (local aspects) often represent elements forming a complex and intricate image; but abstracting from them and instead attending only to the main global features (colors, contrasts, alignment of elements as if blurring out the details) can reveal a larger, often simpler “composition” with a different interpretation (see Figure 1C).

In this study, we capitalized on one kind of ambiguous image to investigate the behavioral and neural correlates of the alleged perceptual states, such as that described in the passage from Hurlburt (1993) where two alternative interpretations of an image are simultaneously perceived. We considered behavioral and brain correlates that simultaneous percepts should exhibit if they are a distinct perceptual state and not, for example, a transitional state or merely an artifact of the language employed to describe them. At the behavioral level, we examined percept duration and switch rate: we first expected that simultaneous states would be as stable as the unambiguous “simple” perceptual states, and that this would be at least partly consistent between participants, in that it would manifest on a group level analysis. We additionally investigated whether the simultaneous percept could be described as an extended transition state by determining if it is most often flanked by the two other percepts. At the brain level, we considered differences in blood-oxygen-level-dependent (BOLD) activity as a function of a perceptual state: we chose ambiguous images that depicted faces and landscapes, to capitalize on the known pattern of BOLD activity in face- and landscape-specific regions, namely fusiform face area (FFA, Kanwisher et al., 1997), occipital face area (OFA, Gauthier et al., 2000) and parahippocampal place area, (PPA, Epstein et al., 1999), respectively. All ambiguous figures fell into the same category (global vs. local processing) thus allowing us to analyze all images together. Several prior studies motivated our approach. BOLD signal levels in FFA and PPA can track the contents of visual awareness in a binocular rivalry setting (Tong et al., 1998), and BOLD signal in FFA increases when the Rubin’s vase figure is interpreted as two faces, rather than a vase (Andrews et al., 2002; Hesselmann et al., 2008). Finally, BOLD signal in PPA increases even when viewing simple line drawings depicting landscapes (Walther et al., 2011). Together, these results suggest a plausible modulation of levels of BOLD signal within FFA, OFA and PPA that correlates with perception.

We selected participants that reported to be able to see two percepts simultaneously in an online survey, and asked them to continuously report their subjective experience, while seeing a bistable image. Our assumption was that the phenomenological differences between the alleged perceptual states should manifest in objective measures of behavior and brain activity. Concretely, we assumed that if the simultaneous percept is a distinct stable percept and not just an unstable transient state, its reported duration would be comparable to that of the two main percepts. Instead, if the simultaneous percept were better described as a transitional state, its reported durations would be much shorter than the two main percepts. Further, some studies have shown individual stability in percept duration (or its inverse, the switch rate), suggesting that this could be considered an individual trait (Kleinschmidt et al., 2012). Therefore, we explored the relationship between the percept durations in all three perceptual states. Furthermore, if the simultaneous state is not just a long transition between two other percepts, then the simultaneous percept that follows one reported percept (e.g., *face*) should in turn not be followed more often than chance by the other percept (e.g., *landscape*).

Further, we sought to characterize brain activity during the simultaneous perceptual state. We hypothesized that BOLD signal levels in FFA, OFA and PPA would be consistent with subjective reports. Concretely, we expected higher activity in FFA and OFA while participants reported seeing the *face* percept, and higher PPA activity during reported *landscape* percepts. Finally, we hypothesized that, if the simultaneous percept is different from the two main percepts (namely, *face* and *landscape*), BOLD signal levels in FFA, OFA and PPA would be distinct for the *simultaneous* percept as compared to the two main ones.

## Materials and Methods

### Participants and Recruitment

We first conducted a short online survey with the double aim of: (a) obtaining statistics from a large sample from the population; and (b) identifying potential study participants who reported to be able to see two percepts simultaneously in an ambiguous image. In the survey, we explained that when seeing ambiguous figures, some people have a preference for seeing one or the other percept, and others have no preference at all. As a cover story, and to avoid biasing responses, we simply stated that we were interested in which “perceptual type” each person was. We did not mention in the survey that we would invite participants based on their responses. In the survey, participants saw six bistable images, and answered two questions for each image. The first question asked directly about the preferred percept. Possible answers were “I see the two figures alternating”; “I see the two figures mostly alternating”; “I see the two figures mostly simultaneously” and “I see the two figures simultaneously”. We also included the alternative “I see only one figure” for cases in which participants could not identify the two alternative interpretations. The second question asked about perceptual effort. Possible answers were: “It is easier for me to see the two figures alternating”; “It is mostly easier to see the two figures alternating”; “It is mostly easier to see the two figures simultaneously” and “It is easier for me to see the two figures simultaneously”. Again, we included the alternative “I see only one figure”.

We distributed a link to the screening survey through the mailing lists of Psychology students of two German universities. We received 282 responses (229 female, 53 male, *mean ± SD*: 23 ± 5 years) in this screening stage. The majority of women that responded to our survey mirrors the majority of women registered as Psychology students in the Universities where we distributed the link. We invited only those participants that scored at least 7 out of 12 points to participate in the experiment (this meant that they had reported that they saw two figures “simultaneously” or “mostly simultaneously” in more than half of the images presented). Seventeen healthy participants accepted to take part (11 female, age ± *SD*: 28.5 ± 4 years). All participants were right handed and had no recent history of psychiatric or neurological disease. This study was carried out in accordance with the recommendations of Ethics committee of the German Psychological Society (DGPs) with written informed consent from all subjects. All subjects gave written informed consent in accordance with the Declaration of Helsinki.

### Stimuli and Responses

For the fMRI scanning, we selected 14 bistable images (consisting in artistic paintings and drawings) in which the alternating percepts corresponded to faces and landscapes (see Figure 1B for examples). None of these images had been presented in the online survey. Further, all participants confirmed in a debriefing after the scanning session that they had never seen the images before.

In the fMRI data analysis (see below), we compared BOLD signal between all three reported percepts, for each participant and irrespective of the image presented. Because the percept dominance was determined by each participant and not experimentally, we could not ensure an even distribution of all three perceptual durations for each image. Thus, and to prevent any systematic errors due to biases by the low-level properties of any particular image that could have dominated any specific percept, we matched the contrast and luminosity of all rivalry images with the SHINE toolbox (Willenbockel et al., 2010a,b). We presented all images using the Psychophysics Toolbox (Brainard, 1997) running in MATLAB 2012b (the Mathworks). We projected the stimuli onto a rear-projection screen mounted inside the scanner bore, at approximately 78 cm from the participants’ eyes. We monitored the manual responses continuously using a Covilex response box (Covilex, Magdeburg, Germany).

### MRI Data Acquisition Parameters

We acquired images on a 3 T Magnetom Trio MRI scanner system (Siemens Medical Systems, Erlangen, Germany) using a 12-channel radiofrequency head coil.

We collected structural images using a three-dimensional T1-weighted magnetization prepared gradient-echo sequence (MPRAGE) based on the ADNI protocol^1^ (repetition time (TR) = 2500 ms; echo time (TE) = 4.77 ms; TI = 1100 ms, acquisition matrix = 256 × 256 × 176, flip angle = 7°; 1 × 1 × 1 mm voxel size). We asked participants to keep their eyes closed during the structural image collection.

We collected functional images using a T2*-weighted echo planar imaging (EPI) sequence sensitive to BOLD contrast (TR = 2000 ms, TE = 30 ms, image matrix = 64 × 64, FOV = 216 mm, flip angle = 80°, voxel size 3 × 3 × 3 mm, 36 axial slices, interleaved order, gap = 0.6 mm).

### Functional Localizer Runs

To localize FFA, OFA and PPA in each participant, we did two localizer scanning runs. In each run we presented eight series of 12 images. Each series of images contained either neutral faces (Grosbras and Paus, 2006) or landscapes. We fully randomized the order of both the images within each series, and the series within each run. We presented each image for 1.3 s with a fixation cross between them of 0.2 s. Participants did a 1-back task during the localizer run. Between runs we presented a baseline image of either a fixation cross or a phase-scrambled image for 15 s.

### Multi-Stable Perception Task

Participants completed two 13.5-min scanning runs of a multi-stable perception task, adapted from classical binocular rivalry tasks requiring continuous report (e.g., Carmel et al., 2010).

In total, we presented 14 different bistable images (see Supplementary Information for details, some examples are displayed in Figure 1C), separated by 30-s baseline period displaying either a fixation cross or a phase-scrambled bistable image. Participants viewed each image for a total of 90 s (Figure 1A). Above the images (on the top right and left corners of the screen), the words “FACE” and “LAND” appeared in gray. Participants used their right hand to hold down a key in the button box corresponding to either percept (*face* or *landscape*, respectively), and held down both keys if they saw the two percepts simultaneously. One or both of the words (“FACE” and/or “LAND”) were highlighted while participants held down the corresponding keys to provide feedback for their responses. We asked participants to avoid focusing on only one of the percepts, and instead to balance the total amount of time across all three percepts. Importantly, we also instructed participants to report as faithfully as possible the percept that they really perceived in that very moment. To increase the rivalrous aspect of the images, we split the 90-s presentation time into two 45-s periods. During the first 2 s of each of these periods, we presented two attentional cues. These cues could not be taken as emphasizing either the *face* or the *landscape* interpretation of the image. Instead, as the example in Figure 1B illustrates, a cue could be pointing to either be an eye or a mountain according to the specific percept. With this instruction, we aimed to prevent participants from simply focussing on different parts of an image (e.g., the sky for *landscape* viewing and the eye region for *face* viewing). We asked participants to focus on these regions, and try to interpret them as any one of three options: exclusively part of the face, exclusively part of the landscape, or as part of both simultaneously. Importantly, the attentional instruction cue was only displayed for 2 s and disappeared afterwards to avoid obstructing the images. Participants were asked to continue focussing on the cued feature of the image even after the attentional cue had disappeared.

We defined three conditions based on subjective report, namely *face*, *landscape* and *simultaneous*. Thus, the total duration of each trial was determined by each participant’s behavior.

### Functional Magnetic Resonance Imaging (fMRI) Data Processing and Analyses

We analyzed the fMRI data with the SPM8 package^2^ running on Matlab version R2012b. We excluded the first four volumes of each EPI series from the analysis to allow the magnetization to approach a dynamic equilibrium. We applied slice-time correction and realignment to all EPI sets. A mean image for all EPI volumes was created, to which we spatially realigned individual volumes by means of rigid body transformations. We co-registered the structural image with the mean image of the EPI series. We normalized the structural T1 image to the Montreal Neurological Institute (MNI) template and applied the normalization parameters to the EPI images to ensure an anatomically informed normalization. Finally, we smoothed images with a kernel of 8 mm full-width at half maximum (FWHM).

We ran statistical analyses at the subject level using a general linear model (GLM). In the localizer runs, we modeled each stimulus presentation as a discrete event, with a fixed duration given by the duration of each image. In the multi-stable perception task, we modeled each self-reported percept event (each trial) as a discrete event, with a duration given by the total trial duration. The resulting vectors were convolved with a canonical hemodynamic response function (HRF) and its temporal derivatives to form the regressors in a design matrix. We used a high-pass filter of 128 s to remove low-frequency drifts in the time series data. We also included in the GLM realignment parameters in all 6 dimensions to account for variance associated with head motion. Statistical parameter estimates were computed separately for each voxel for all columns in the design matrix at the within-participant level.

We followed the Group-Constrained Subject-Specific (GSS) approach (Julian et al., 2012; Nieto-Castañón and Fedorenko, 2012) to define subject-specific regions of interest. We used the parcels for right and left FFA and PPA derived by Julian et al. (2012). We then obtained participant-specific ROIs by overlaying the parcels to each participant’s corresponding contrast map (i.e., *faces* > *landscapes*, for FFA and OFA, and *landscapes* > *faces* for PPA), thresholded at *p* < 0.001 (uncorrected). Finally, we used the Marsbar toolbox (version 0.43^3^) to extract BOLD signal estimates from the ROIs. We calculated the 10-s time course by subdividing this period into five distinct epochs of 2000 ms (1 TR) and used five finite impulse response regressors to estimate the BOLD signal change for each epoch independently.

## Results

### Online Screening

**Figure 2:**
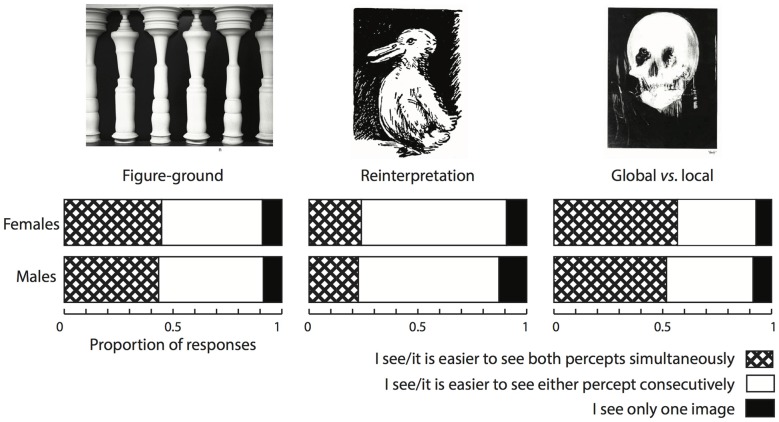
Population screening results. Results from a total of 282 responses. A large part of the screened population reported that they could see the two percepts simultaneously especially for images in the “Figure-ground” and “Global vs. local categories”.

Figure 2 shows the percentages of responses for each of the three image categories presented in the online questionnaire (*figure-ground*, *reinterpretation* and *global* vs. *local*, including two images each). Multiple sources of variability have been related to individual differences in the properties of bistable perception. Albeit in a different paradigm of gender perception in point-like walkers, Schouten et al. (2010) reported gender differences in biases in bistable perception. Because one of the aims of the online screening was to select and invite participants for the fMRI study, we examined responses from men and women separately to explore potential gender differences that would be relevant for fMRI participant recruitment. Across all included images, 39% of men and 41% of women replied that they saw, or that it was easier to see the two possible interpretations simultaneously, whereas 51% of men and 49% of women replied that they saw either percept sequentially. A *χ*^2^ test revealed no significant gender differences in the proportion of participants that reported seeing the two interpretations simultaneously ($\chi_{\left(1\right)}^{2}$ = 0.094, *p* = 0.759), so we did not consider participants’ gender when selectively inviting them for the fMRI study.

Taken at face value, these results show that an important part of the student population that we surveyed reported to be able to entertain two percepts simultaneously, especially in the case of those images that we included in the “Figure-ground” and in the “Global vs. Local” categories. This was less strongly so when the alternation between the two percepts required a complete reinterpretation of the image.

The results of this short online survey do not provide any details about the phenomenology of the simultaneous percept so, to better understand this alleged simultaneous perceptual state, we measured BOLD signal activity in a selected sample of participants while they viewed ambiguous images. In line with the survey results, the ambiguous images that we selected as stimuli depicted one stimulus (a face) when processed globally and a different stimulus (a landscape) when processed locally.

### Rivalry Task: Behavioral Results

In the fMRI scanner, participants reported continuously which was the dominant percept (*face*, *landscape*, or *simultaneous*). We first considered the behavioral durations of each percept.

**Figure 3:**
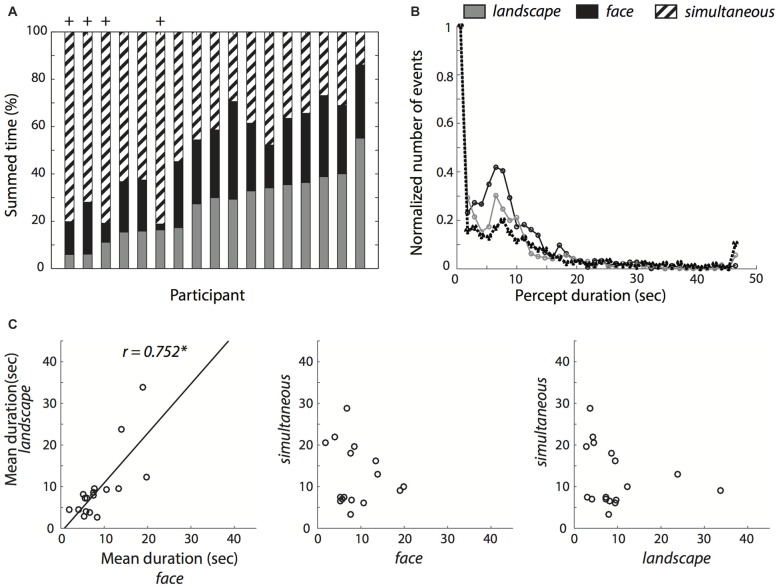
Reported percept durations. **(A)** Total summed percept durations for each participant relative to the summed duration for all percepts. **(B)** Histograms of percept durations normalized by the number of events in the bin with the greatest number of events for each perceptual condition. The *simultaneous* condition showed a similar duration profile as the single *face* and *landscape* conditions. **(C)** Correlations between mean percept durations within participants. The solid black line represents the least squares fit. *Indicates a significant correlation at *p* < 0.05.

Figure 3A shows the summed duration of each percept, for each participant. We included all participants in the behavioral analyses, but excluded four participants (marked with a + symbol in Figure 3A) from the subsequent fMRI analyses because the summed duration of at least one of the percepts represented less than 10% of the summed experiment duration, which could lead to biased estimates (Wager et al., 2005). Figure 3B shows the distribution of durations for each percept, aggregated for all participants. The distribution of the *simultaneous* percept is very similar to the distribution of the *face* and *landscape* percepts, indicating that it is not just a short-lived transition state, but that, on the contrary, it is at least as stable as the other two percepts.

Because percept durations have often shown to be stable and trait-like (Kleinschmidt et al., 2012), we examined the mean duration of each percept: on average, participants indicated to have seen the simultaneous percept longer (*M* = 12.29 s, *SD* = 7.26 s) than only the landscape (*M* = 9.40 s, *SD* = 7.97 s) or only the face (*M* = 8.74 s, *SD* = 5.04 s) in the pictures. These differences were not statistically significant (one-way analysis of variance (ANOVA); (*F*_(1.27,20.31)_ = 1.38, *p* = 0.262, *partial* *η*^2^ = 0.08, Greenhouse-Geisser corrected). We also examined the number of occurrences of each percept. Here, participants showed more occurrences of the *simultaneous* percept (*M* = 60.0, *SD* = 27.89) than only the *landscape* (*M* = 40.88, *SD* = 15.78) or the *face* percepts (*M* = 38.24, *SD* = 15.90). A one-way ANOVA revealed that the differences were statistically significant (*F*_(1.98,31.65)_ = 16.63, *p* < 0.001, *partial* *η*^2^ = 0.51). Follow-up *t*-tests revealed that this difference was driven by the greater number of occurrences of the *simultaneous* percept (*simultaneous* vs. *landscape*: *t*_(16)_ = 4.49, *p* < 0.001, *d* = 1.09; *simultaneous* vs. *face*: *t*_(16)_ = 5.20, *p* < 0.001, *d* = 1.26), but not between the *face* and *landscape* percepts (*t*_(16)_ = 0.68, *p* = 0.51, *d* = 0.16).

We then asked if simultaneous percepts are merely transitional. Concretely, we calculated for each participant the proportion of *simultaneous* percept flanked by two different percepts, relative to all simultaneous percepts flanked by any two percepts. We compared this to the proportion corresponding to chance (1/2). We found that the proportion was slightly but reliably above chance level (*M* = 0.611, *SD* = 0.134, one-sample *t*-test *t*_(16)_ = 3.415, *p* = 0.004, *d* = 0.828). We note however that very short percept durations (i.e., 0.2 s) were relatively overrepresented in the data (Figure 3B). We therefore repeated the analysis, this time excluding trials under 0.2 s Here, we found no evidence to suggest that the proportion was significantly different from chance level (*M* = 0.467, *SD* = 0.226, one-sample *t*-test *t*_(16)_ = −0.551, *p* = 0.589, *d* = −0.134). This suggests that while short *simultaneous* percepts were often transitional, this was not necessarily the case for the longer-lasting *simultaneous* percepts.

When examining the relationship between the percept durations with a Pearson correlation test, there was a clear correlation between the *face* and *landscape* conditions (*r* = 0.752, *p* < 0.001) but we found no evidence for a correlation between the *simultaneous* and *face* (*r* = −0.160, *p* = 0.540) or *simultaneous* and *landscape* conditions (*r = −0.228*, *p* = 0.379). Further, we did a Fisher *r*-to-*z* transformation to statistically test the differences in correlations. We found that the correlation between the mean *face* and *landscape* durations differed significantly from both the correlation between *face* and *simultaneous* conditions, (*z* = 3.01, *p* = 0.003) and from the correlation between the *landscape* and *simultaneous* conditions (*z* = 3.2, *p* = 0.001).

### Rivalry Task: fMRI Results

**Figure 4:**
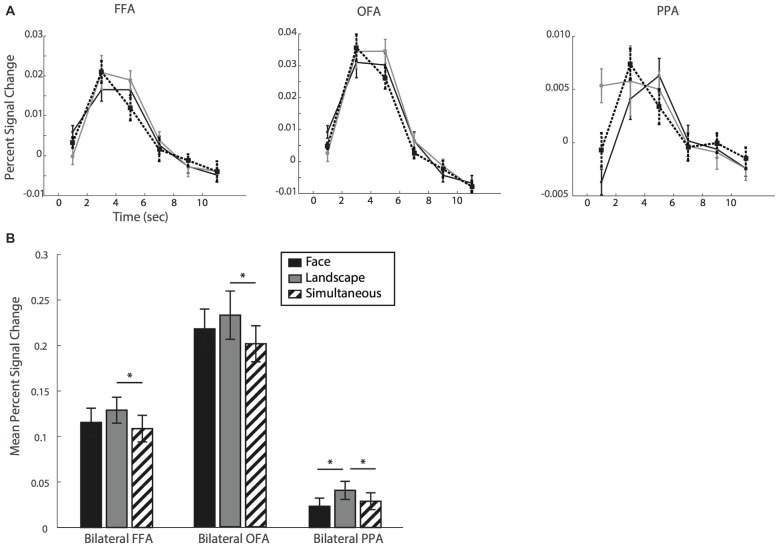
**(A)** Time course of the mean percent signal change for fusiform face area (FFA), occipital face area (OFA) and parahippocampal place area (PPA). **(B)** Mean percent signal change relative to baseline and averaged over a time window of 4–6 s. *Indicate *P* < 0.05, error bars show standard error.

Finally, we quantified BOLD signal levels in FFA, OFA and PPA, areas known to show selectively stronger BOLD activity with attention to faces or landscapes. We extracted the BOLD signal in each condition relative to baseline in the three ROIs. Figure 4 shows the relative signal change estimates. (Note that, to avoid computing biased estimates, we discarded four participants—see Figure 3A. The following results are therefore based on 13 participants). We show in Figure 4A the time courses of the BOLD signal corresponding to each of the three reported percepts (*face*, *landscape* and *simultaneous*) for each of the three ROIs.

In a planned comparison and as a positive control, we first examined BOLD signal levels in PPA and FFA associated with the *face* and *landscape* conditions. We ran our statistical analyses on the mean percent signal change over a window of 4–6 s as a summary measure (Figure 4B). The PPA ROI showed the expected pattern. Namely, the *landscape* percept was associated with stronger BOLD signal levels than the *face* percept (*t*_(12)_ = 3.148, *p* = 0.008, *d* = 0.872). This suggests that participants were able to accurately identify and report their subjective perceptual state. Turning to our main condition of interest, we examined the BOLD signal level for the *simultaneous* condition. We found that it was significantly lower than for *landscape* (*t*_(12)_ = −2.511, *p* = 0.027, *d* = −0.696) though not different than the *face* condition (*t*_(12)_ = 1.554, *p* = 0.146, *d* = 0.431).

FFA and OFA, instead, did not show the expected pattern. We expected the *face* percept to show the strongest levels of BOLD signal in these face-selective regions. Instead, we found no differences between *face* and *landscape* conditions (with *t*_(12)_ = −1.884, *p* = 0.084, *d* = −0.523 for FFA and *t*_(12)_ = −1.626, *p* = 0.130, *d* = −0.451 for OFA). When we compared BOLD signal for *simultaneous* and *face* percepts in these regions, we found no differences relative to the *face* condition (FFA: *t*_(12)_ = −1.169, *p* = 0.265, *d* = −0.324; OFA: *t*_(12)_ = −1.686, *p* = 0.118, *d* = −0.467); but significant differences between the *simultaneous* and *landscape* conditions (FFA: *t*_(12)_ = −4.522, *p* < 0.001, *d* = −1.254; OFA: *t*_(12)_ = −2.898, *p* = 0.013, *d* = −0.804).

## Discussion

The different possible interpretations of ambiguous images are typically taken to be mutually exclusive, as it is assumed that the multiple percepts compete for conscious access. In our survey and the present experiment, we showed that this might not always be the case. In an initial population screening we found that, when prompted, a large proportion of the population (39% of men and 41% of women) reported that they were able —and sometimes found it easier—to *simultaneously* entertain the two percepts; rather than experiencing them in alternation. To better understand these phenomenological reports, we explored the objective correlates of the alleged subjective state. In a group of selected participants we explored the behavioral and physiological bases for the reported *simultaneous* perceptual state, and compared them to those of the single percepts. As a way to track the brain activity associated with the perception of—and attention to—each of the two percepts, we used bistable images that included a face and a landscape, which were expected to elicit stronger BOLD signals levels in FFA and OFA, and in PPA respectively. In an fMRI task, we asked participants to view these bistable images, and to continuously report their perceptual state. We defined three conditions (*face*, *landscape* and *simultaneous*), based on each participant’s subjective report.

We sought to identify any potential objective measures that would distinguish the *simultaneous* condition from the other two, supporting the phenomenological reports, which identify it as a distinct perceptual state. We use the fMRI data as objective evidence to back the subjective and phenomenological claims.

We first examined the reported durations for each of the percepts. Most participants were able to follow our instructions to split the viewing time approximately equally between the three conditions. Histograms of the percept durations showed that the *simultaneous* percept is at least as stable as the *face* and *landscape* percepts, suggesting that it is not just a transitional, short-lived perceptual state. And, while short-lasting reports of the *simultaneous* percept were more often than chance flanked by two different percepts (i.e., preceded by face and followed by landscape or vice versa), this was not the case for the longer-lasting reports of the *simultaneous* percept. This suggests that the *simultaneous* state is not necessarily the transition between the other two “simple” percepts. Further, we examined the correlations between the mean duration of each percept, for each participant (see Figure 3C). We found a significant correlation between the mean durations of the *face* and *landscape* percepts, in line with previous reports that the dynamics of spontaneous perceptual alternations have trait-like characteristics (Kanai et al., 2010; Kleinschmidt et al., 2012; van Loon et al., 2013). Interestingly however, we found no significant correlations between the mean percept duration between the *simultaneous* condition and *face* or *landscape* conditions. The correlation between *face* and *landscape* mean durations was significantly different from the correlations between simultaneous and both face and landscape conditions. This result was against our hypothesis of a correlation between all three perceptual states. However, because the distribution of durations of the simultaneous percept was comparable to that of the other two percepts and the mean duration of the simultaneous state was even longer than for the other two perceptual states, we do not think that this result can be taken as strong evidence that the simultaneous percept is simply transitional. Instead, we note that we instructed participants to try to interpret the images in all three states during the scanning session. Thus, although admittedly none of our participants reported this at debriefing, we speculate that our explicit instructions might have led to a greater degree of voluntary control in the transitions to and from the *simultaneous* percept, and participants attempting to sustain the *simultaneous* percept for longer, leading to a breakdown in the correlations with the *face* and *landscape* percepts, which depend more strongly on mechanisms of spontaneous perceptual alternation (Meng and Tong, 2004).

Finally, we inspected BOLD signal levels in ROIs defined within FFA, OFA and PPA, for each of the three conditions. With the fMRI data analysis, we aimed to find an objective measure that could back up our participants’ claim, that they perceived the two possible interpretations *simultaneously*.

First, as we expected, the *landscape* condition in PPA was associated with stronger levels of BOLD signal activity than the *face* condition. This is consistent with participants’ subjective reports and validates our approach. The *simultaneous* condition was associated with BOLD signal levels in PPA lower than those for the *landscape* condition, but not different from those for the *face* condition.

The levels of activity in FFA and OFA across conditions, on the other hand, were not informative. BOLD signal in these two ROIs did not vary as expected with the reported control conditions (*face* and *landscape*): BOLD signal levels for *face* reports were no different than those for *landscape*. According to our participants’ subjective reports, we explain this result *ad hoc*: at debriefing all participants spontaneously reported that the faces depicted in the images were very difficult to ignore. Admittedly however, this is a speculation that we cannot support with behavioral data, because we found no evidence to suggest that *face* percepts occurred more often or lasted longer than *landscape* percepts. However, we argue that subjective reports are to be trusted, including those at debriefing. Hence, taken together, the subjective reports during the task, at debriefing and the behavioral results during the task suggest that participants may have found it more effortful to attend to the landscape only and completely ignore or suppress the *face* percept. As a consequence, they might have been only partly successful at doing so. Thus, periods during which *landscape* percepts were reported could have also included perceived faces. In line with this suggestion, faces are more quickly and more accurately recognized than landscapes or houses, even when presented upside down Diamond and Carey (1986). This is consistent with the idea that face recognition is an obligatory detection process (Tsao and Livingstone, 2008) and faces are processed in an automatic and holistic fashion: that is to say faces are represented as non-decomposed wholes rather than the combination of independent components (like the eyes, nose and mouth; Tanaka and Farah, 1993; Farah et al., 1998; Logothetis, 2000; Palermo and Rhodes, 2007). Especially activation in the FFA indicates that the face as a whole is detected and not its low-level stimulus features (Tsao and Livingstone, 2008). This could explain why BOLD signal activation in FFA was high across conditions: neurons selective for holistic face recognition in the FFA may have continuously detected the face in the pictures at all times, which is why it was difficult for participants to entirely disregard it.

### Differences between the Simultaneous State and Piecemeal Perception

We argue that the simultaneous state allegedly experienced by our participants is phenomenologically and physiological different from the mixed states or piecemeal perception typically observed in many cases of bistable perception (Knapen et al., 2011). These mixed states, consistently reported by experimental participants, are clearly transition states that are flanked by two “simple” percepts. We found no evidence that this was the case for the simultaneous percept. Also tellingly, mixed percepts are often described as a state of “mixed dominance” (Brascamp et al., 2015). In our view, this description underscores the notion of competition because while no single stimulus dominates the entire visual field, the two alternative stimuli share the space and are alternatively dominant in separate local regions of the visual field. In contrast, according to our participants’ reports—or, perhaps fairly, according to our best understanding of or participants’ reports—, dominance was not exclusive. Participants were allegedly able to interpret the same point in space within the visual field as belonging to *both* a face and a landscape. No participant spontaneously reported any piecemeal perception as it has been described in, for example, traveling waves of piecemeal perception in binocular rivalry paradigms. We thus take the strong view that the phenomenology that we describe here is different from that reported before in mixed states. Admittedly however, stronger evidence for this view could only come from specifically probing participants to describe the differences between the two ways of simultaneous perception.

### Determinants of the Properties of Bistable Perception

The online survey that we first conducted revealed that there is natural variability within the normal population on the properties of bistable perception. This observation goes in line with the literature highlighting differences between individuals in the dynamics of bistability. Inter-individual differences have been related to molecular, structural and functional factors (for reviews see Kleinschmidt et al., 2012; and Scocchia et al., 2014). For instance, at the molecular level differences between mean perceptual duration (or switch rate) in auditory and visual rivalry have been associated with genetic differences in the dopamine and serotonin receptors (Kondo et al., 2012) and with differences in GABA concentration measured with MR spectroscopy (van Loon et al., 2013, but see Sandberg et al., 2016 for Bayesian analyses of the same effects, suggesting that the some of the correlations reported were overestimated or even false positives). Individual differences in brain structure have been found to correlate with multiple cognitive functions (Kanai and Rees, 2011), and bistable perception is no exception. For example, the speed of visual traveling waves has been associated with cortical surface area in visual areas V1 and V2 (Genç et al., 2015); and biases in bistable motion have been related with microstructure of callosal segments (namely, radial diffusivity, in this case presumably reflecting axon diameter) connecting motion-sensitive areas of the human MT/V5 complex (Genç et al., 2011). Further, structural characteristics of the superior parietal lobe (SPL) have been causally related to the rate of perceptual alternations in a rotating structure-from-motion stimulus (Kanai et al., 2010). Finally, the characteristics of bistable perception have also been described and manipulated at the functional level. Importantly, this level of analysis has revealed that perceptual switch rate and other characteristics cannot always be considered as a stable individual trait, as the relationships to brain structure might suggest. For example, tACS-induced gamma (but not alpha) activity increased the rate of perceptual switches in a structure-from-motion task (Cabral-Calderin et al., 2015), as did intoxicating levels of alcohol intake on the duration of piecemeal perception during a binocular rivalry task (Cao et al., 2016). Attentional effects can also have a profound impact on biases in binocular rivalry (Pearson et al., 2008; Dieter et al., 2016).

Given the multiple levels at which differences in brain structure and function can be translated into overt differences in the properties of bistable perception, it is possible that the ability to entertain two percepts simultaneously is related to measurable properties of brain structure. With this study we highlight the possibility that the perception of simultaneous states might serve as an additional parameter of interest in the understanding of bistable perception.

### Reliability of Subjective Report

It has been argued that introspection cannot be trusted as a reliable source of information: subjective report can be incomplete and inaccurate (Nisbett and Wilson, 1977; Schwitzgebel, 2008), alter the object of introspection (Seli et al., 2013) and may add spurious monitoring mechanisms on top of the first order cognitive processes of interest (Guggisberg et al., 2011). Many of these points were compellingly illustrated in a recent experiment of binocular rivalry (Frässle et al., 2014). Here, we present the flip side of the argument. Despite the clear limitations of introspection as a method, ignoring the subjective experience of participants altogether, and favoring a strictly behaviorist approach might lead to overlooking certain fundamental aspects of cognitive functioning, as we illustrate with the results we present here. We started from participants’ subjective report, and explored the objective evidence that supported its veracity. We found that the behavioral and neurophysiological correlates of the reported subjective states gave first evidence to the notion that entertaining two percepts simultaneously can be a distinct state. Therefore, we highlight the importance of a balanced contribution of different experimental approaches to the literature (Callard and Fitzgerald, 2014).

### Limitations

We should point to several important limitations of these data. Perhaps the strongest limitation is that they do not suffice to distinguish a true *simultaneous* percept from a quick alternation between the two single percepts. In other words, it is in principle possible that what participants perceive to be the coexistence of two percepts was in fact a very rapid alternation between two single percepts. When asked this question explicitly, our participants consistently maintained that the term “simultaneous” was a more accurate description of their subjective experience. Conducting interviews with participants trained in describing their pristine experience (Hurlburt and Heavey, 2006), through which participants acquire tools to observe and describe their subjective states might be a way to further understand the phenomenology of the simultaneous perception states that we identified.

Our data are specific in two important ways that limit their generalizability. First, we tested only one kind of bistable stimuli where the two percepts resulted from either local or global processing. We selected these stimuli because they contained faces and landscapes as alternating percepts, both of which have known brain correlates. These stimuli would then allow us to identify objective correlates of the subjectively reported percepts, thus validating the subjective report. Importantly however, these stimuli are not representative of all kinds of bistable perception (as discussed further above, see Hasson et al., 2001). Our conclusions may also not hold true for other, also widely used stimuli where rivalry does strictly happen, as is perhaps the case for the Necker cube (Necker, 1832), the structure-from-motion illusion (Nawrot and Blake, 1989) or the kinetic depth effect (Wallach and O’Connell, 1953). Further studies could extend this approach to other forms of stability and ask the important question of generalizability of these results.

Second, because in this study we were specifically interested in studying the simultaneous percept, we investigated their behavioral and brain correlates only in participants that reported to experience it and did not include a “control group” of participants that did not experience the simultaneous percept. While this comparison would not have been informative in understanding the nature of simultaneous percept alone, it could have informed the interpretation of the fMRI results. As we described above, several studies have reported inter-individual differences in perceptual exclusivity. Hence, it is in principle possible that the putative automatic face processing and accompanying enhanced BOLD activation in the face-selective areas FFA and OFA was not a consequence of the stimulus material but an idiosyncrasy of this preselected subgroup. Finally, our sample of participants, explicitly selected for high levels of simultaneous perception, reported similar durations of the *simultaneous* and the alternative “simple” percepts. It is possible that a different sample of participants for whom entertaining two percepts simultaneously is possible but difficult would have revealed a qualitatively different pattern of the duration subjective reports. Thus, these results could be extended in interesting ways by including a comparison to a control group to explore, for example, whether there are any differences in brain structure that could account for this difference in the available perceptual states.

## Author Contributions

EF, MB, YW and SK designed the experiment; wrote the article. MB and YW did the online survey. EF programmed the experimental task. EF, MB and YW collected the data; analyzed the data.

## Conflict of Interest Statement

The authors declare that the research was conducted in the absence of any commercial or financial relationships that could be construed as a potential conflict of interest.

## Footnotes

^1^www.adni-info.org

^2^http://www.fil.ion.ucl.ac.uk/spm/

^3^http://marsbar.sourceforge.net/
